# Integrated analysis of single cell and spatial transcriptomics revealed a metastasis mechanism mediated by fatty acid metabolism in lymph nodes of head and neck cancer

**DOI:** 10.3389/fimmu.2025.1614498

**Published:** 2025-08-13

**Authors:** Jinru Weng, Jiajie Mao, Yixing Li, Jun Zhao, Xiaolin Nong

**Affiliations:** ^1^ College of Stomatology, Hospital of Stomatology, Guangxi Medical University, Nanning, Guangxi, China; ^2^ Department of Stomatology, Yiwu Central Hospital, Yiwu, Zhejiang, China; ^3^ Stomatology Hospital, School of Stomatology, Zhejiang University School of Medicine, Zhejiang Provincial Clinical Research Center for Oral Diseases, Key Laboratory of Oral Biomedical Research of Zhejiang Province, Cancer Center of Zhejiang University, Engineering Research Center of Oral Biomaterials and Devices of Zhejiang Province, Hangzhou, China; ^4^ Guangxi Key Laboratory of Oral and Maxillofacial Rehabilitation and Reconstruction, Guangxi Medical University, Nanning, Guangxi, China

**Keywords:** fatty acid metabolism, LGALS1, metastasis, single-cell RNA sequencing, spatial transcriptome

## Abstract

**Introduction:**

Head and neck squamous cell carcinoma (HNSCC) was a common malignant tumor, and its recurrence and metastasis during treatment were the main factors affecting the patient's prognosis. This study aimed to explore the evolutionary mechanisms during HNSCC metastasis.

**Methods:**

This study collected single-cell RNA sequencing (scRNA-seq), spatial transcriptome (ST) data, and fatty acid metabolism-related genes from public databases for pseudo-chronological, differentiation, cell interaction, and pathway analysis. In vivo and in vitro experiments were conducted to study the expression of LGALS1 and its role in HNSCC cells.

**Results:**

Three specific subclusters in the tumor metastasis process were identified, including primary tumors, transitional tumors, and metastatic tumors. During tumor evolution, fatty acid metabolism was upregulated, and active fatty acid metabolism involving LGALS1 was related to HNSCC cell metastasis. Knocking down LGALS1 significantly inhibited the proliferation, migration, and lymph node metastasis ability of HNSCC cells and changed the expressions of E-cadherin, Snail, and PPARγ at the protein level.

**Discussion:**

This study described metabolic changes during HNSCC dissemination and revealed the critical role of metastatic tumors in the mechanism of HNSCC metastasis by regulating fatty acid metabolism.

## 1 Introduction

Head and neck squamous cell carcinoma (HNSCC) ranks among the most prevalent malignancies globally. Despite extensive research, the precise etiology of HNSCC remains elusive. However, several well-documented risk factors, including smoking, human papillomavirus (HPV) infection, and alcohol, have been identified as significant contributors to the development of this cancer. Although substantial advancements have been achieved in conventional treatment modalities, the highly invasive nature and propensity for early lymph node metastasis of HNSCC continue to pose significant challenges (1). Therefore, tumor invasion and metastasis are key factors affecting patient survival and prognosis.

Single-cell RNA sequencing (scRNA-seq) is a cutting-edge technology that enables high-throughput sequencing analysis of the genome, transcriptome, and epigenome at single-cell resolution. It has emerged as a powerful tool with significant applications in cancer research, biology, and the study of disease onset and progression (2). Compared with single-cell sequencing technology, Spatial transcriptome (ST) can combine the location information of cells in tissue distribution with gene expression information to understand cell heterogeneity in a spatial context. Recent research has revealed that metastatic lesions from the same patient often share a close clonal relationship, while primary and metastatic tumors remain distinct, as observed in breast and liver cancers (3, 4). Two prevailing hypotheses explain the origin of metastatic tumors: one suggests gradual evolution, where metastasis occurs in the late stages of tumor progression; the other proposes parallel evolution, positing that tumor cell dissemination happens early in progression and that primary and metastatic tumors evolve concurrently (5). Given these complexities, the integration of scRNA-seq and spatial transcriptomics holds great promise for deepening our understanding of the cellular heterogeneity and pathological mechanisms underlying HNSCC.

Metabolic reprogramming is increasingly recognized as a crucial mechanism by which tumors maintain their malignant biological behavior. Beyond the well-documented alterations in glucose metabolism, fatty acid metabolism (FAM) has also emerged as a vital process in lymph node metastasis (6). Compared with primary tumors, a more pronounced accumulation of fatty acids has been detected in lymph node metastatic tumors. In the lipid-rich lymph node environment, metastatic tumor cells prefer fatty acids over glucose as their primary fuel for energy production. Many cancer cells tend to upregulate key enzymes of fatty acid metabolism to support rapid proliferation through *de novo* lipid synthesis (7–9). Lectin galactoside-binding soluble 1 (LGALS1) is a β-galactoside-binding protein that plays a significant role in lipid metabolism (10). It promotes adipocyte differentiation and lipid accumulation by activating peroxisome proliferator-activated receptor γ (PPARγ) in adipocytes. In leukemia, LGALS1 upregulates the expression of genes involved in lipid uptake (such as CD36) and *de novo* lipogenesis (such as PPARγ, FASN, and ACC), thereby enhancing lipid accumulation (11). Although interventions targeting FAM have been shown to inhibit the progression of solid tumors, its role in lymph node metastasis of HNSCC further exploration (12, 13).

In this study, we integrated scRNA-seq and spatial transcriptomics analyses for the first time to unravel the three specific clusters of head and neck cancer in tumor metastasis, and the mechanisms of FAM in tumor evolution. We also elucidated the role of LGALS1, a pivotal gene, in the metastasis of HNSCC through comprehensive *in vitro* and *in vivo* experiments, as well as immunochemistry. This study offers innovative insights into the heterogeneity and molecular underpinnings of HNSCC, laying the groundwork for the formulation of targeted therapeutic strategies for this cancer.

## 2 Materials and methods

### 2.1 Data acquisition, processing, and annotation

The single-cell RNA sequencing (scRNA-seq) data was acquired from the GSE181919, and GSE188737 datasets downloaded from the Gene Expression Omnibus (GEO) database (https://www.ncbi.nlm.nih.gov/geo/).

The scRNA-seq and ST data were processed using the R package Seurat (version 4.2.0). The cells contained over 500 expressed genes and a mitochondrial UMI rate below 35% passed the cell quality filtering and mitochondrial genes were removed in the expression table. To calculate the subset of genes that show high cell-to-cell variability in the dataset, we processed the data using the “vst” method in the FindVariableFeatures algorithm, which by default selects the top 2000 highly variable genes for downstream analysis. Subsequently, these genes were scaled before conducting a principal component analysis (PCA). To remove the batch effect, which may affect the accuracy of single-cell analysis, we applied the batch effect correction analysis by the Harmony package (14) with the default parameter. Finally, to achieve a refined cell clustering, we applied the FindClusters function to classify cells into different clusters according to the recommendations of Seurat, with a default resolution value of 0.8. The RunUMAP() function was performed for visualization. We calculated the marker genes by FindAllMarkers function with the Wilcox rank sum test algorithm. The R package SingleR (15), with the reference paper was used to infer the cell of origin of each of the single cells independently and identify cell types.

### 2.2 Inferring copy number variation from scRNA-seq data

According to the default parameters, the InferCNV package (version: 1.20.0) (16) was applied to evaluate the copy number variation (CNV) of each cell for each region on the chromosome based on the amount of gene expression in the single-cell transcriptome data. The CNV levels of epithelial cells were calculated, and normal epithelial cells were applied as the reference.

### 2.3 Cell trajectory analysis

CytoTRACE was used to capture, smoothed, and calculated the expression levels of genes that were highly correlated with the single-cell gene counts of scRNA-Seq data. After the CytoTRACE algorithm was completed, each cell was assigned a score and given Wilcoxon rank-sum test statistical analysis. The trajectory analysis was performed using the Monocle2 package (version 2.28.0) (17) to reveal the cell-state transitions. Based on the pseudotime analysis, branch expression analysis modeling (BEAM) analysis was applied for branch fate determined gene analysis. CytoTRACE v0.3.2 package was applied to predict the differentiation score and plotted on the monocle trajectories. CytoTRACE v0.3.2 package was applied to predict the differentiation score and plotted on the monocle trajectories.

### 2.4 Cell communication analysis

To enable a systematic analysis of different cell communication pattens, we applied cell communication analysis based on the Cellchat package (version 1.6.1) (18), which was designed for inferring and analyzing intercellular communication networks from scRNA-seq data.

### 2.5 Identification of differentially expressed genes

To identify differentially expressed genes among the three specific subclusters in the GSE181919 dataset, the function FindMarkers with the Wilcox rank sum test algorithm was used under the following criteria: 1, lnFC > 0.25; 2, p-value <0.05; and 3, min. pct > 0.1. The plots were visualized using the ggplot2 and VennDiagram package.

### 2.6 Gene function analysis

Gene ontology (GO) analysis is a common method for large-scale functional enrichment studies, and the Kyoto Encyclopedia of Genes and Genomes (KEGG) pathway analysis were carried out to predict the possible courses and pathways. The R package ClusterProfiler was used to perform GO and KEGG annotation analysis of data. The item screening criteria were p-value < 0.05 and FDR value (qvalue) < 0.25. The padj correction method was Benjamini-Hochberg. To find which functional pathways work in specific cell clusters, GSVA package (version 1.30.0) (51) was used to perform gene set enrichment analyses based on H dataset (version 7.2) from MsigDB (http://www.gsea-msigdb.org/gsea/msigdb/) and calculated scores for pathway activity values in each cell.

### 2.7 ST analysis

The ST data was also acquired from the GSE208253 and GSE220978 datasets downloaded from the GEO database. For the quality control of ST data, spots with more than 10% mitochondrial genes or fewer than 200 detected gene counts were discarded. Then, the other steps are basically the same as those for scRNA-seq data analysis. The SPOTlight (19) was applied to deconvolute cell types within scRNA-seq. SpaCET (20) was used to explore cancer cell states and FAM sources.

### 2.8 Metabolic pathway analysis

The RNA-seq data were acquired from 566 HNSCC specimens downloaded from the Cancer Genome Atlas (TCGA) (https://portal.gdc.cancer.gov/) and 117 normal specimens acquired from the Genotype-Tissue Expression database (https://www.gtexportal.org/), comprising their pertinent clinical information, which included the vital condition, age, gender, tumor grade, and pathological stage of patients. Single-sample GSEA (ssGSEA) scores were calculated for 85 KEGG metabolic pathways based on gene expression levels. The activity difference of KEGG metabolic pathways between lymph node metastasis and non-metastasis was measured by two-sided Wilcoxon rank-sum test. P values were adjusted for multiple testing using the Benjamini–Hochberg method. The ssGSEA analysis was performed in R package GSVA. P value <0.05 was considered as statistical significance.

### 2.9 Immunochemistry

The head and neck microarrays were from Bioaitech (cat. no. HN058Oc01; Shanxi, China). Sample information was detailed in **Supplementary Table S1**. Then deparaffinized with xylene and gradient alcohol, repaired by microwave with sodium citrate (PH6.0) for 20 minutes. Incubate with 3% H_2_O_2_ avoiding light for 15 minutes, add LGALS1 primary antibody (1:200; cat. no. CY7610; Shanghai Abways Biotechnology Co., Ltd.), and incubate overnight at 4°C. After rinsing with PBS, add anti-rabbit IgG secondary antibody (Boster, Wuhan, China) and incubate at 37°C for 30 minutes. After washing with PBS, add DAB chromogenic reagent dropwise, stain the nucleus with hematoxylin, and mount the slide after dehydration.

### 2.10 Cell line and cell culture

Human HNSCC cell lines HSC4 and CAL27 were purchased from the American Type Culture Collection (ATCC). All cells were cultured in Dulbecco’s Modified Eagle’s medium (DMEM) with high glucose (GIBCO, U.S.A.) containing 10% fetal bovine serum (FBS, Biological Industries, Israel) and 100 U/ml penicillin/streptomycin (GIBCO, U.S.A.) at 37°C in a humidified atmosphere of 5% CO_2_. The LGALS1 shRNA were purchased from GeneCopoeia Co., Ltd. (Guangzhou, China).

### 2.11 Cell transfection

The HSC4 and CAL27 cells, at 30% confluency, were seeded in 6-well plates containing serum-free medium. LGALS1 shRNA or the negative control shRNA was transfected into these cells using polybrene (Beyotime, Beijing, China). Forty-eight hours post-transfection, the cells were subjected to puromycin selection to establish stable cell lines with the transfection. LGALS1 overexpression plasmid was purchased from GenePharma (Shanghai). The HSC4 and CAL27 cells were seeded on six-well plates according to the instructions of each manufacturer. When the cells grew to 70-90%, the transfection reagent Lipofectamin2000 was added. After incubation in the incubator for 5 hours, the transfection solution was discarded and replaced with complete culture medium. The efficacy of the knockdown and overexpressed was confirmed through western blot analysis.

### 2.12 Western blot assay

The RIPA lysate (Boster, Wuhan, China) was used to lyse and extract the cell protein. Separate the same amount of protein by SDS-PAGE electrophoresis with 3 μL per well on 8% or 12% gel, and transfer to PVDF membrane. Block the PVDF membrane with 5% skimmed milk at 37°C for 1 hour and incubate the primary antibody overnight at 4°C. The next day, TBST was washed 4 times/15min, and the secondary antibody (1:5,000; cat. no. BA1056 and BA1058, respectively; Boster, Wuhan, China) was incubated for 1 hour at 37°C. According to the manufacturer’s instructions, using ECL kit (cat. no. P0018FS; Beyotime Institute of Biotechnology) to visualize the protein bolt. Analyze the results with Image J software. The names of Primary antibodies are as follows: β−actin (1:5,000; cat. no. AB0011; Shanghai Abways Biotechnology Co., Ltd.). LGALS1 (1:1000; cat. no. CY7610; Shanghai Abways Biotechnology Co., Ltd.). Snail (1:1000; cat. no. BY0174; Shanghai Abways Biotechnology Co., Ltd.). E-cadherin (1:1000; cat. no. 20874-1-AP; Wuhan Sanying Co., Ltd.). PPARγ (1:1000; cat. no. EPR18516; Shanghai Abcam Biotechnology Co., Ltd.).

### 2.13 Cell proliferation assay

According to the manufacturer’s instructions, the Cell Counting Kit-8 (CCK-8; Boster, Wuhan, China) was performed to evaluate the ability of proliferation. The transfected HSC4 and CAL27 cells were seeded in 96-well plates with 2 × 10^3^ cells per well containing 100 μl of complete medium, respectively. After 24, 48, 72, 96h of culture, 10 μl of CCK-8 reagent was added into each well and the 96-well plates continued to be incubated in a humidified incubator for 1 h, and the optical density (OD) for each well was finally detected at 450 nm with a microplate reader.

### 2.14 Transwell assays

The transfected HSC4 and CAL27 cells were seeded in the upper chamber with serum-free medium and add complete medium containing 20% FBS to the bottom chamber. After 48 h of incubation at 37°C, the cells were fixed with 4% paraformaldehyde for 15 min. The cells on the upper side of the membrane were wiped off with a cotton swab, followed by staining with 0.1% crystal violet for 10 min at room temperature. Five randomly selected microscopic fields were counted using an upright fluorescence differential interference contrast microscopy system.

### 2.15 Wound healing assay

The transfected HSC4 and CAL27 cells were seeded in 6-well plates with 3×10^5^ per well containing 1 mL DMEM/high glucose medium supplemented with 10% FBS. When the cells were 95% fused, a serum-free medium was used instead of complete culture, and a sterile pipette tip was used to form a linear wound. Record the results.

### 2.16 Animal assay

All animal experiments were approved by the Ethics Committee of Zhejiang University. Eight male Nu/Nu nude mice, 5~6 weeks old, weighing 18~22g, were purchased from Charles River Co., Ltd. (Beijing, China). All animals were housed in a well-ventilated room with a 12-h light/dark cycle (room temperature: 25 ± 2°C, humidity: 50% ± 10%) and given standardized food and water. After a 7-day acclimatization period, the mice were randomly divided into two groups: shCtrl and shLGALS1. The HSC4 cells in the logarithmic growth phase were prepared into a cell suspension with a concentration of 4×106 cells/mL, and then, 0.1 mL of the cell suspension was injected subcutaneously into the tail vein of the nude mice in the two groups. After 30 days, the mice were killed by cervical dislocation. Subsequently, the lung tissues were fixed using 4% paraformaldehyde, and paraffin sections were prepared for H&E staining to confirm the presence of metastasis.

### 2.17 H&E staining

The lung tissues of mice were harvested and washed with PBS, followed by fixation in 4% paraformaldehyde. After dehydration through an ethanol gradient, the tissues were made transparent by dissolving the alcohol and paraformaldehyde in xylene. The samples were then embedded in melted paraffin at an elevated temperature and sectioned into slices with a thickness of 4μm. The sections were dewaxed and rehydrated through a gradient of xylene and ethanol, stained with hematoxylin for nuclei and eosin for cytoplasm, and then dehydrated and made transparent through a gradient of ethanol and xylene. After drying, the sections were mounted with neutral resin. Finally, the sections were observed under a microscope, images were captured and analyzed.

### 2.18 Statistical analysis

All statistical analyses were performed in GraphPad Prism 8.0 software and R software (4.2.2). P < 0.05 is considered significant. (*P < 0.05, **P < 0.01).

## 3 Result

### 3.1 Annotation of cell clusters and tumor subclusters analysis

**Figure 1:**
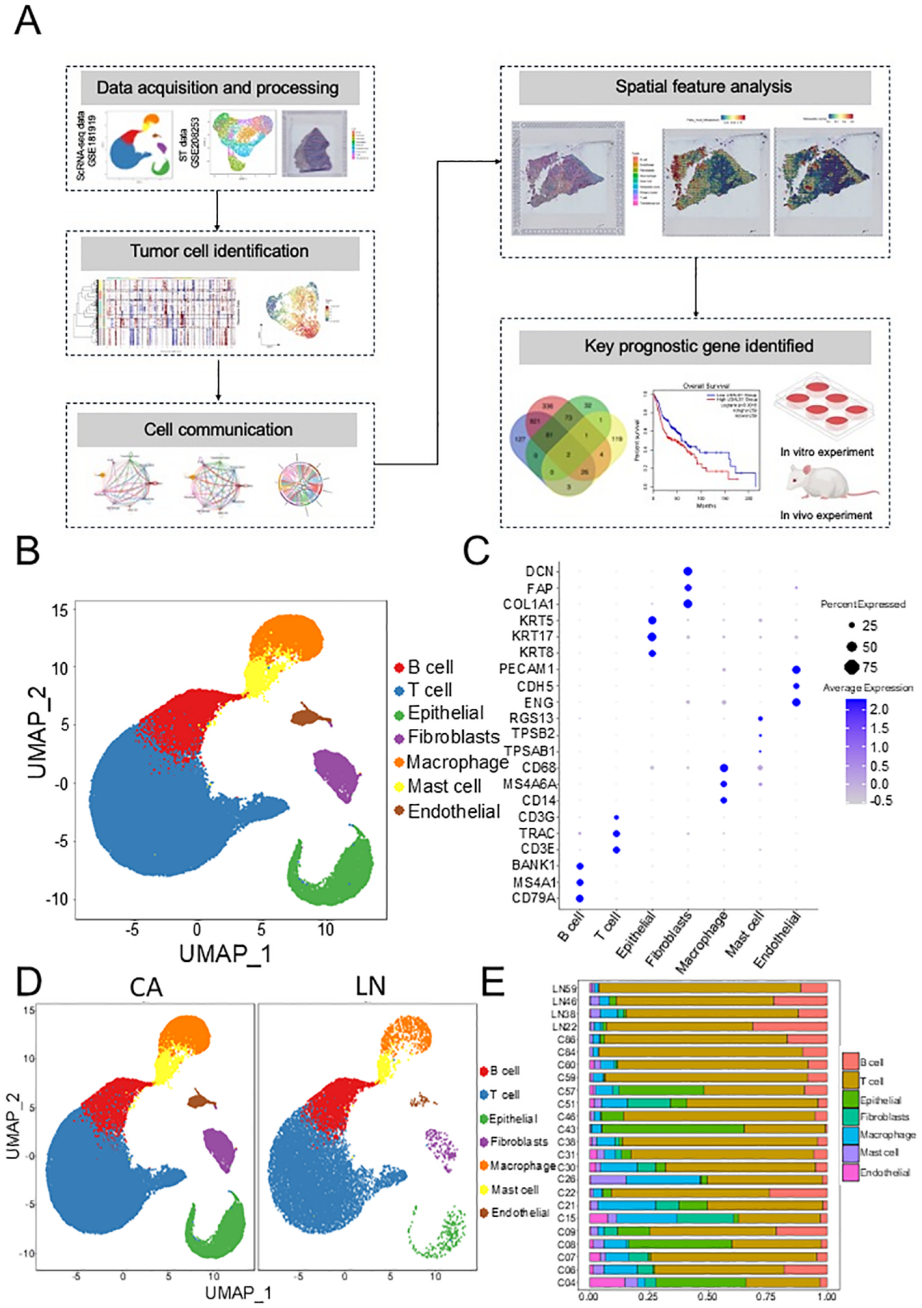
Investigation the association between primary and lymph nodes in HNSCC based on GSE181919 dataset. **(A)** Graphic overview of this study design. **(B)** Scatter plot of the annotated cell clusters. **(C)** Bubble plots of the marker genes expressed in the major cell types. **(D)** Scatter plot of annotated cell clusters in different groups. **(E)** The composition of cell subpopulations between the different groups. HNSCC, Head and neck squamous cell carcinoma.

In this study, the workflow was exhibited in **Figure 1A**. The GSE181919 dataset, including 20 primary HNSCC tissue samples and 4 lymph node metastasis samples, was employed to explore the metastasis mechanism in HNSCC. After data quality control, we annotation 7 cell types (**Figure 1B**), according to their canonical markers: B cells (CD79A, MS4A1, BANK1), T cells (CD3E, TRAC, CD3G), Epithelial (KRT5, KRT8, KRT17), Fibroblasts (COL1A1, FAP, DCN), Macrophage (CD14, MS4A6A, CD68), Mast cell (TPSAB1, TPSB2, RGS13), Endothelial (ENG, CDH5, PECAM1) (**Figure 1C**). These 7 cell types were detected in almost every patient (**Figure 1D**); however, there were differences in composition between primary and metastatic sites (**Figure 1E**, **Supplementary Figure S1**), especially epithelial cells. This suggested that in addition to the regulatory role of immune cells in the TME, tumor cells themselves had also evolved.

**Figure 2:**
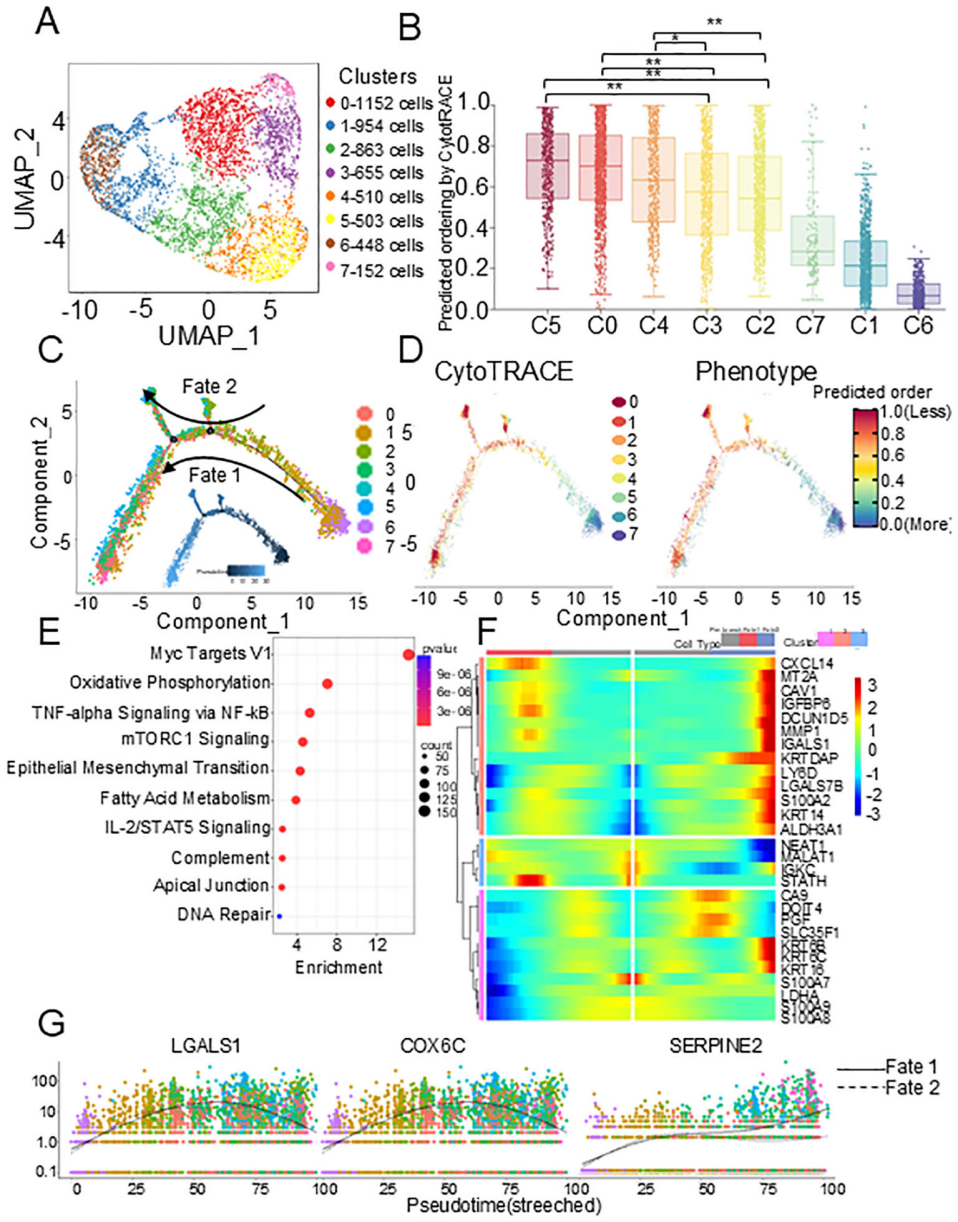
Metastatic epithelial cell characteristics identified by scRNA-seq. **(A)** UMAP plot of epithelial cells showing 8 clusters. **(B)** Box plots of the CytoTRACE scores in 8 epithelial cell clusters. P values were calculated using two-sided Wilcoxon rank-sum test with Benjamini–Hochberg correction. **(C)** Monocle trajectory of all epithelial cells identified two distinct cell fates colored by cluster. **(D)** CytoTRACE scores to derive trajectory. **(E)** Bubble plot showing the top 10 pathways. **(F)** The heat map of differential expression genes in different cell types of the pseudotime trajectory. **(G)** Dot plots of dynamic expression of key genes in different pathway. scRNA-seq, single-cell RNA sequencing. *p < 0.05; **p < 0.01.

Since HNSCC is derived from epithelial cells, we then performed high-resolution UMAP analysis and re-cluster the epithelial cells into 8 subclusters (**Figure 2A**). To distinguish malignant from nonmalignant clusters, the inferCNV package was employed to evaluated the CNV levels of each epithelial cell cluster. As expected, CNV levels in tumor epithelial cells were significantly higher than those in reference cells (normal epithelial cells) (**Supplementary Figure S2A**). Among all epithelial cell clusters, according to the CytoTRACE score, the two-sided Wilcoxon rank-sum test showed that the scores of C0, C4, and C5 were significantly higher than those of other clusters, which was also verified by the two-sided Wilcoxon rank-sum test of CNV score (**Figure 2B**, **Supplementary Figure S2B**), indicating that tumor cells exist in different states. Therefore, to identify the evolutionary process of HNSCC tumor lineages, we constructed trajectories using Monocle 2.0. The pseudotime trajectory began with C1 and C6 due to their lower CNV levels, and then split into two main branches with C3 and C5 placed at opposite divergent ends as two terminally differentiated cell types (**Figure 2C**). In addition, CytoTRACE also revealed a distinct dedifferentiated subclusters in primary tumor that had high differentiation potential (**Figure 2D**). We postulate that in these tumors, distinct subclusters in the primary tumor showed a more aggressive phenotype, that likely evolved further after nodal dissemination had occurred. Therefore, the tumor clusters were divided into three subclusters, primary tumors (C1, C6), transitional tumors (C2, C3, C7), and metastatic tumors (C0, C4, C5), based on the CNV score and CytoTRACE score.

In metastatic tumors, enrichment analysis based on Molecular Signatures Database (MsigDB) (21) identified the altering pathways during the evolution process. Among the top 10 most enriched pathways, in addition to the classic pathways related to cell proliferation, tumor metastasis, and tumor immunity, we found that the fatty acid metabolism pathway was significantly enriched, indicating that tumor metabolic reprogramming plays an important role in metastasis (**Figure 2E**). The dynamic analysis of gene expression showed that the marker genes in Oxidative Phosphorylation (OXPHOS) (COX6C, ISCA1, COX6A1) and the marker genes in fatty acid metabolism pathway (ECI2, LGALS1, NIP7) showed an up-then-down expression pattern during the early dissemination of malignant epithelial cells. At the same time, marker genes of epithelial-to-mesenchymal transition pathway (CLDN3, SERPINE2) showed an expression pattern of being up-regulated and then tending to be stable, indicating that the process of dissemination is related to metabolism (**Figures 2F-G**, **Supplementary Figures S2C, D**).

### 3.2 Cell-cell interaction between tumor subclusters and immune cells in the TME

**Figure 3:**
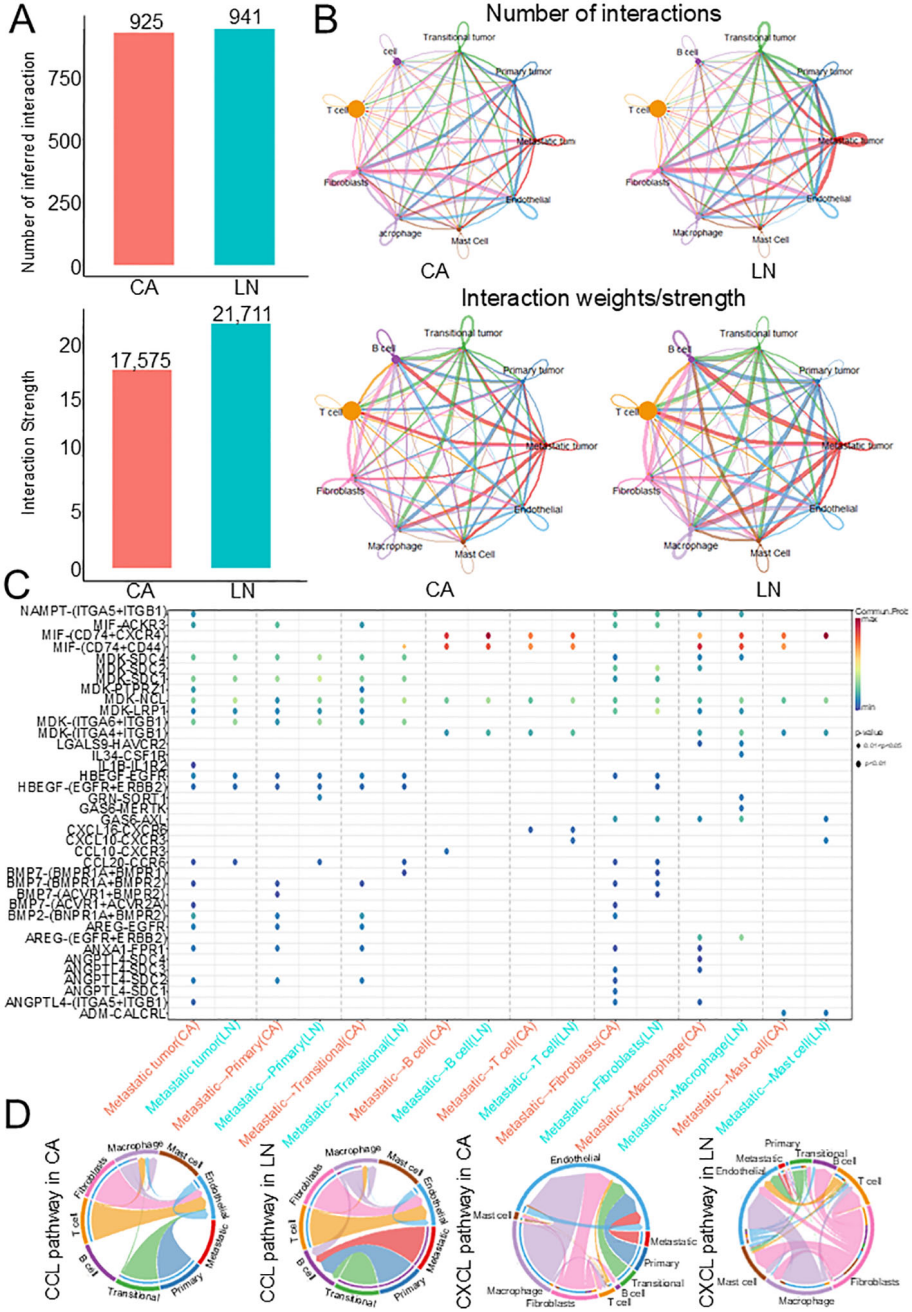
Cell-cell communication analysis in HNSCC. **(A)** Bar plot showing the number and strength of intercellular interactions in both lymph node and tumor. **(B)** Heatmaps of differential number and strength of intercellular interactions between lymph node and tumor. **(C)** Bubble plots of the significant differentially expressed ligand–receptor pairs in the lymph node versus tumor. **(D)** Chord diagrams of the inferred CCL and CXCL signaling networks between primary and lymph nodes. HNSCC, Head and neck squamous cell carcinoma.

To identify the communication between these three tumor subsets and immune cells in the TME, we used CellChat to analyze cell-cell interactions. Although the number of interactions between the major cell types in primary tumors was very similar, the strength of interactions was significantly enhanced in cells with lymph node metastases, and metastatic tumors in lymph node metastases showed stronger interactions with immune cells (**Figures 3A, B**). We found that ligand-receptor pairs between metastatic tumors and immune cells were significantly upregulated in lymph nodes compared with primary tumors (CCL10-CXCR3, IL34-CSF1R, GAS6-MERTK), indicating that these pathways are critical for the immune response to tumors (**Figure 3C**). In addition, we found that CCL and CXCL pathways were particularly active in lymph nodes, and these pathways were involved in cell proliferation, migration and apoptosis (**Figure 3D**). These results suggest that these three tumor subclusters may play key roles in the pathogenesis of HNSCC.

### 3.3 Spatial transcriptomics combined with scRNA-Seq reveals spatial features of FAM in tumor subclusters

**Figure 4:**
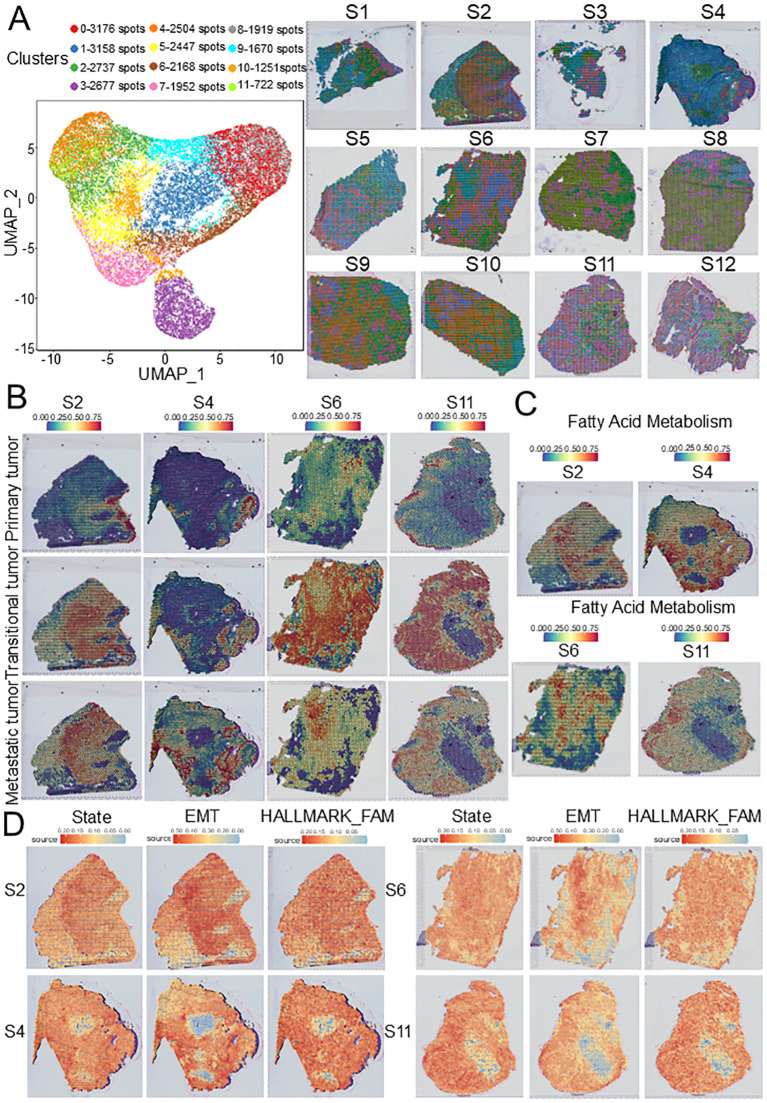
ST characteristics of HNSCC based on GSE208253 dataset. **(A)** UMAP plot of 26,381 spots from 12 samples showing 12 clusters. **(B)** SPOTlight map of overlap between scRNA-seq-identified three tumor subclusters and ST identified spot clusters. **(C)** FAM source feature plots from ST generated using the GSVA. **(D)** Cell states, EMT source, and FAM source feature plots from ST generated using SpaCET. ST, Spatial transcriptomics; EMT, epithelial–mesenchymal transition; FAM, Fatty acid metabolism; HNSCC, Head and neck squamous cell carcinoma.

To further evaluate the spatial distribution of the three tumor subpopulations, we selected GSE208253 dataset for spatial transcriptome analysis. After integrating and normalizing spots from each sample, 26,381 spots from 12 samples were divided into 12 clusters (**Figure 4A**). To describe the spatial characteristics of the three tumor subclusters and other cells, SPOTlight was used to map scRNA data onto ST slides. We found that compared with those without lymph node metastasis, metastatic tumors were significantly increased on ST slides with lymph node metastasis (**Figure 4B**, **Supplementary Figure S3A**), and T cells were significantly concentrated at the boundary between transitional tumors and metastatic tumors (**Supplementary Figure S3B**), indicating that this front area has unique spatial characteristics. Interestingly, the results of GSVA also showed that the FAM pathway was significantly upregulated in the spots of metastatic tumors (**Figure 4C**, **Supplementary Figures S2C, D**), indicating that tumor metabolism has unique spatial characteristics.

To validate the association between the FAM pathway and HNSCC dissemination, the SpaCET was used to evaluate the cell states, epithelial-to-mesenchymal transition (EMT) source, and FAM source in ST slides. We found that in the late stages of the tumor, EMT and FAM in tumor cells increased significantly, and their spatial distribution was consistent with that of metastatic tumors, suggesting that the upregulation of FAM may be closely related to lymph node metastasis and that the evolution from the initial stage to the disseminated stage is parallel to the tumor metabolic pathway (**Figure 4D**, **Supplementary Figure S4**).

### 3.4 Identification and functional analysis of prognostic genes related to FAM

**Figure 5:**
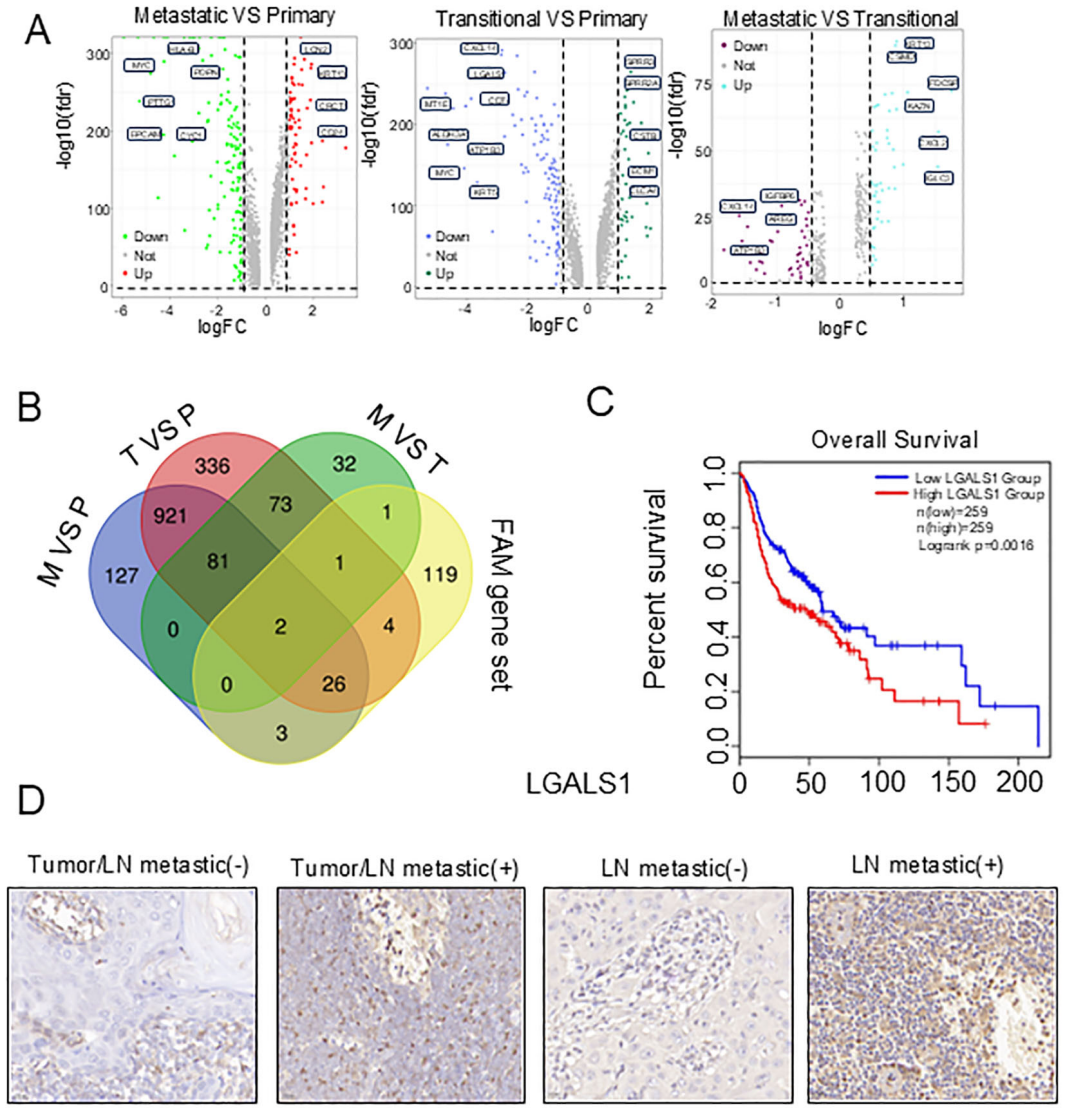
Establishment of FAM-related prognostic hub genes in HNSCC. **(A)** Volcano plots of differentially expressed genes in three tumor subclusters. **(B)** Venn diagram showing the hub gene identified in three tumor subclusters. **(C)** The Kaplan-Meier curve showed that patients with high expression of LGALS1 had a poor prognosis. **(D)** Immunohistochemistry images of LGALS1 expressions in primary tumor with or without lymph node metastasis.

To identify hub genes in the tumor subclusters, we intersected the following three gene sets: genes highly expressed in metastatic tumors (M *vs* P; M *vs* T) and genes highly expressed in transitional tumors (T *vs* P) (**Figure 5A**). Next, we intersected the hub genes with the 152 FAM-related genes and obtained two overlapping genes, LGALS1 and Aldehyde dehydrogenase 3 Family member A1 ALDH3A1 (**Figure 5B**). Subsequently, survival analysis was conducted to ascertain the relations of the expression of LGALS1 and ALDH3A1 with HNSCC prognosis. According to the Kaplan-Meier survival curve, patients with higher LGALS1 expression in HNSCC had shorter overall survival, while ALDH3A1 expression was not significantly related to patient prognosis (**Figure 5C**, **Supplementary Figure S5A**).

### 3.5 Validation of LGALS1 to promote HNSCC cells proliferation, migration, and EMT *in vitro* and *in vivo*

**Figure 6:**
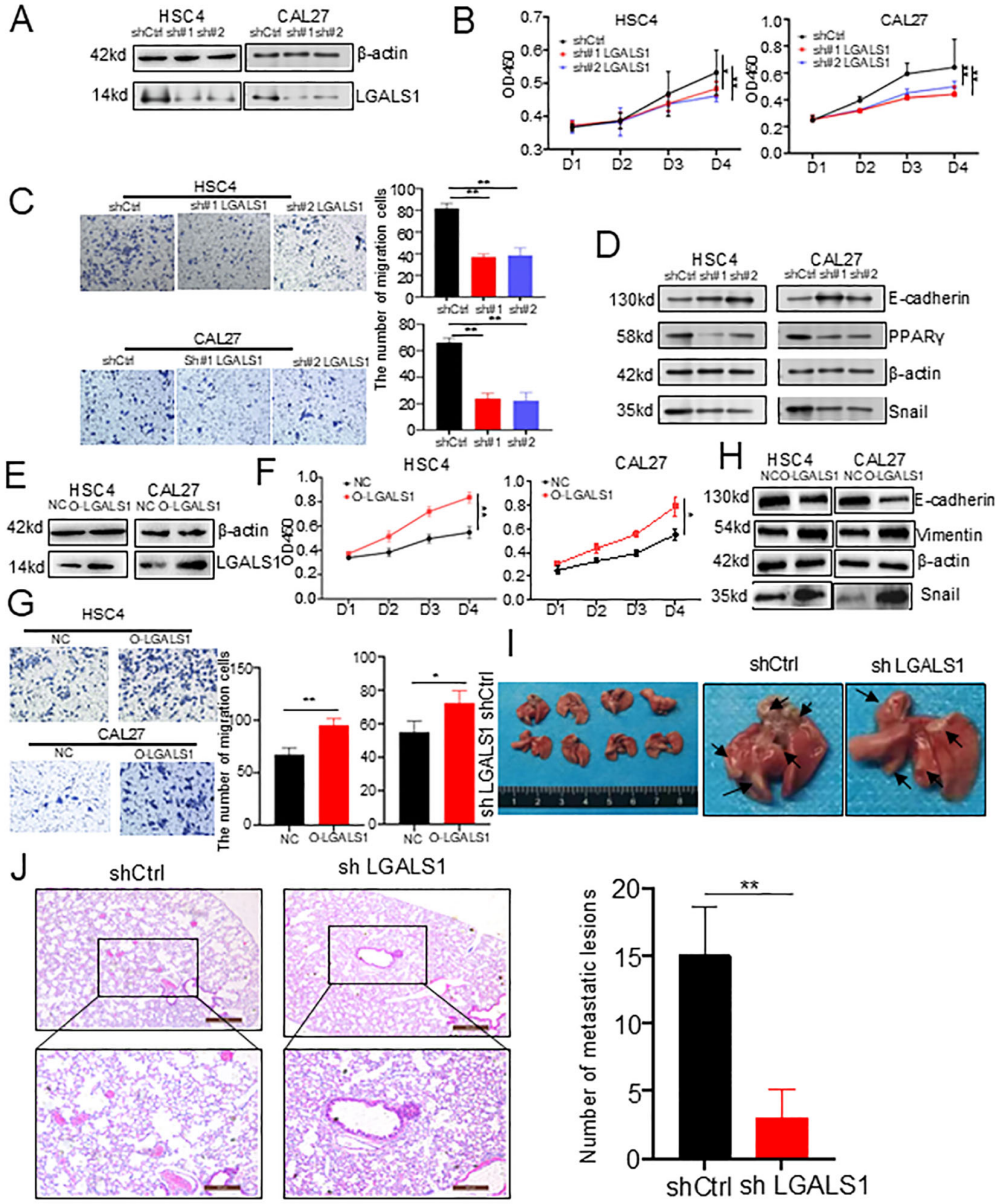
*In vitro* and *in vivo* experiments validated the effect of LGALS1 on HNSCC progression. **(A)** Western bolt showed that LGALS1 was successfully knocked down. **(B)** Line plots showed significantly lower cell proliferation in HSC4 and CAL27 cells after knocking down LGALS1. **(C)** Bar plots showed downregulation of LGALS1 significantly inhibited cell invasion ability of HSC4 and CAL27 cells in trans well assay (right). **(D)** Western bolt showed that low expression of LGALS1 may affect the expression of Lipid or EMT related proteins. **(E)** Western blot showed that LGALS1 was successfully overexpressed. **(F)** Line plots showed significantly higher cell proliferation in HSC4 and CAL27 cells after overexpressed LGALS1. **(G)** Bar plots showed upregulation of LGALS1 significantly increased cell invasion ability of HSC4 and CAL27 cells in trans well assay (right). **(H)** Western bolt showed that high expression of LGALS1 may affect the expression of EMT related proteins. **(I)** Gross images of lung tissues of nude mice in the control group and LGALS1 knockout group. **(J)** The bar plots showed that downregulation of LGALS1 significantly inhibited the invasive ability of HSC4 cells in nude mice (right). EMT, epithelial–mesenchymal transition; HNSCC, Head and neck squamous cell carcinoma; *p < 0.05; **p < 0.01.

To investigate the impact of LGALS1 on tumor progression, we first compared the expression levels of LGALS1 between lymph nodes and primary tumors with positive or negative lymph node metastasis using immunohistochemistry. We observed that the protein expression of LGALS1 was increased in samples with positive lymph node metastasis (**Figure 5D**, **Supplementary Table S2**). To future explore the effect of LGALS1 on cell proliferation, migration, and invasion, we established LGALS1 knockdown and mock transfection of HSC4 and CAL27 cells, Western bolt showed that the protein expression of LGALS1 decreased significantly (**Figure 6A**, **Supplementary Figure S5B**). The results of CCK-8 showed that the proliferation ability of HSC4 and CAL27 cells knockdown LGALS1 was significantly decreased after 4 days of culture, compared with mock transfection cells (**Figure 6B**). Transwell and wound healing results showed that knockdown of LGALS1 significantly reduced their invasion, and migration activities (**Figure 6C**, **Supplementary Figure S5C**). In addition, we found that the expression of E-cadherin was upregulated while Snail and PPARγ were downregulated in LGALS1 knockdown HSC4 and CAL27 cells (**Figure 6D**, **Supplementary Figure S5D**), which suggested that LGALS1 may promote cell migration through upregulating EMT related pathways through lipid accumulation. To further verify the effect of LGALS1 on the malignant behavior of HNSCC, we established LGALS1 overexpression and empty HSC4 and CAL27 cell lines, and verified the transfection efficiency by Western bolt (**Figure 6E**, **Supplementary Figure S5E**). The results of CCK-8 experiment showed that compared with empty cells, the proliferation ability of HSC4 and CAL27 cells overexpressing LGALS1 was significantly improved after 4 days of culture (**Figure 6F**). The results of Transwell experiment showed that overexpression of LGALS1 significantly promoted the invasion ability (**Figure 6G**). Consistent with the aforementioned findings, the overexpression of LGALS1 led to an upregulation of Vimentin and Snail in HSC4 and CAL27 cells. In contrast, the expression of E-cadherin was correspondingly diminished (**Figure 6H**, **Supplementary Figure S5F**). To verify the role of LGALS1 *in vivo*, we selected HSC4 cells that are more prone to tumorigenesis and injected them into the tail vein of nude mice to establish a lung metastasis model. The results showed that compared with LGALS1 knockdown, cancer cell metastases could be clearly seen on the surface of lung tissue of nude mice in the mock group (**Figure 6I**). Histological analysis found that compared with mock transfection, the number of lung metastases in nude mice with LGALS1 knockdown was significantly reduced. Compared with mock transfection, the number of lung metastases in nude mice with LGALS1 knockdown was significantly reduced, suggesting that LGALS1 can significantly promote tumor metastasis *in vivo* (**Figure 6J**).

### 3.6 Upregulation of FAM exhibited associations with lymph node statue in HNSCC patients

**Figure 7:**
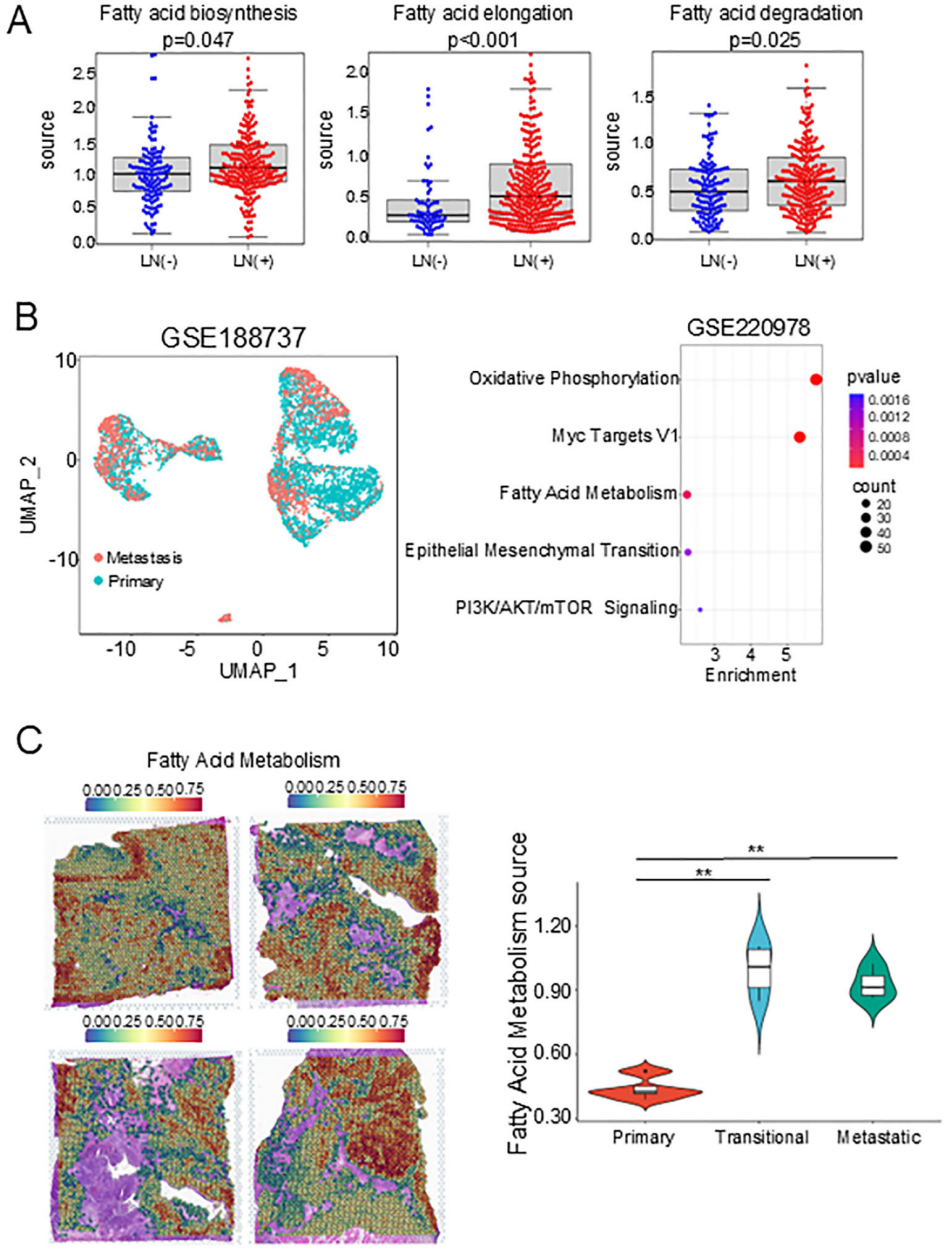
Validation of FAM’s association with lymph node status in early disseminated head and neck cancer. **(A)** Dot plots showing the signature scores of FAM pathways in Lymph Node Status statue. **(B)** UMAP plot of epithelial cells. **(C)** Bubble plot showing the top 5 pathways in GSE188737 dataset. **(D)** FAM score feature plots in GSE220978 dataset. FAM, Fatty acid metabolism. FAM, Fatty acid metabolism. **, P<0.01.

To verify the role of the FAM pathway in tumor dissemination, we performed single sample gene set enrichment analysis (ssGSEA) on some patients in the Cancer Genome Atlas (TCGA) and Genotype-Tissue Expression (GTEx) databases. Patients with lymph node metastasis had significantly higher scores in fatty acid synthesis, fatty acid elongation, and fatty acid degradation compared with patients without lymph node metastasis (**Figure 7A**), suggesting that upregulation of FAM may be associated with lymph node metastasis and was related to lipid synthesis. At the same time, we selected the GSE188737 and GSE220978 datasets for further validation, which included scRNA-seq data of HNSCC in primary tumors and metastases to lymph nodes and ST data of oral squamous cell carcinoma. As expected, FAM and EMT pathways were enriched in metastatic lymph nodes (**Figure 7B**), and the two clusters with the highest FAM in the four ST samples were distributed in metastatic tumors and transitional tumors (**Figure 7C**, **Supplementary Figure S6**), which was consistent with our results. In summary, we found that three subclusters existed in tumor cells and exhibited metabolic features of upregulated FAM and played a crucial role in tumor dissemination.

## 4 Discussion

Metastasis of cervical lymph nodes is an independent factor affecting the prognosis of HNSCC. The more lymph node metastasis there is, the worse the treatment and prognosis of the patient. In addition, tumor heterogeneity affects a series of biological functions, such as hormone response, energy metabolism, and immune response. However, most studies have focused on the characteristics of the immune microenvironment of HNSCC, and few have focused on specific cell clusters in the process of lymph node metastasis. In this study, we identified three tumor subclusters by integrating scRNA-seq and ST and elucidated their spatial distribution in tissue samples and their interactions with other cells. In addition, we found that tumor cells promote transformation through FAM during the evolution of head and neck cancer dissemination. Our study helps to further understand the bioenergetic state of disseminating cells after seeding, providing valuable insights into the complex mechanisms of this highly heterogeneous malignancy.

The formation of metastasis is a relatively rare phenomenon in tumors, as cancer cells must surmount numerous environmental hurdles to successfully establish themselves in other organs (22). Cancer cells evolve through genetic, epigenetic, and transcriptomic changes to gain the ability to colonize distant sites (23). Accumulating evidence indicates that dynamic metabolic alterations in metastatic cells confer a more invasive phenotype on cancer cells (24, 25). In this study, we investigated specific subclusters of tumor cells in early-stage disseminated HNSCC and their metabolic changes during evolution, based on the GSE181919 dataset. A study by Puram et al (26). reported that stromal cells and immune cells had consistent expression programs in HNSCC primary tumors and lymph node metastases, while related features such as cell cycle, epithelial differentiation, and EMT were different between different tumor cells. Our research has revealed that there are three specific subclusters within tumor cells, and that cell state, degree of differentiation, and EMT exhibit dynamic changes during early dissemination. Meanwhile, cell-to-cell communication plays a vital role in head and neck cancer. Neighboring cells, including the three tumor subclusters and immune cells, facilitate dissemination (27, 28). In this study, we determined that the CCL and CXCL pathways were highly active between metastatic tumor cells and immune cells using CellChat. The CCL and CXCL signaling pathways promote the infiltration and metastasis of tumor cells by regulating the migration of immune cells and the immune response of TME, becoming an important molecular mechanism in the process of tumor metastasis (29, 30).

To explore the driving factors that lead to tumor evolution, we found that FAM is the most distinctive pathway besides cell cycle and EMT through the MsigDB database. Recent studies have shown that obesity is associated with cancer progression, metastasis formation, and mortality in multiple cancer types, including prostate cancer, melanoma, and breast cancer, and that lipids are functionally implicated in several steps of the metastatic cascade (31, 32). Increased fatty acid uptake and lipid accumulation, as well as increased expression of fatty acid metabolism-related genes (such as fatty acid transporters), promoted cancer cell invasion, migration, and metastasis (33). Another study showed that metastatic and non-metastatic oral squamous cell carcinoma cell lines differ in lipid metabolism (34). *De novo* fatty acid synthesis was also implicated in the ability of cancer cells to colonize distant organs. In hepatoma cells, genetic knockout of CD147 reduced fatty acid synthesis by disrupting the Akt/mTOR signaling pathway and upregulating peroxisome proliferator-activated receptor α (PPARα), thereby increasing proliferation and metastasis formation in cell lines and mouse models (35). Our study suggested that fatty acid synthesis and metabolism could promote the transformation of metastatic cancer cells during tumor dissemination, and once colonized, the metastatic tumors showed more malignant properties, which was also confirmed in ST, and TCGA or GTEx databases.

This study focused on the metabolism evolvement in the early disseminated HNSCC cancer cells. Through dynamic analysis of gene expression, we found that the expression of genes such as LGALS1, ECI2, and SERPINE2 showed an up-regulated trend in the early dissemination of tumors. Li et al. found that LGALS1 was upregulated in highly invasive oral cancer cells, and in oral cancer tissue specimens, increased LGALS1 expression was associated with tumor progression and lymph node metastasis (36). ECI2, a protein present in both mitochondria and peroxisomes, is thought to be involved in the β-oxidation of polyunsaturated fatty acids. Chen et al. found that inhibiting the expression of ECI2 could reduce ether lipid-mediated IL-8 expression, thereby leading to reduced neutrophil recruitment and reduced neutrophil extracellular trap formation, inhibiting colorectal cancer (37). SERPINE2 was a member of the serine protease inhibitor superfamily. It had been reported that it could activate the EMT process by regulating the expression of E-cadherin, Snail and ZEB through BMP4, thereby inducing tumor metastasis (38).

In this study, LGALS1 was identified as the most important FAM-related hub gene for evaluating the prognosis of HNSCC. Cao et al. found that silencing the LGALS1 gene in cancer-associated fibroblasts (CAFs) inhibited CAF-induced tumor cell migration and invasion *in vitro*, as well as tumor formation *in vivo*, suggesting that blocking the LGALS1 gene could be a potential approach for treating liver cancer (39). Nambiar et al. found that LGALS1 promoted the aggregation of myeloid-derived suppressor cells (MDSCs) in the pre-metastatic microenvironment through the NF-κB signaling axis, thereby triggering CXCL2-mediated enhanced MDSC migration and promoting the metastasis of head and neck cancer. This was consistent with our research results. We found that sh-LGALS1 can significantly inhibit the malignant behaviors of HNSCC cells, including proliferation and migration. *In vivo* experiments also confirmed that knocking down LGALS1 inhibited lymph node metastasis of tumors in nude mice. We also found that LGALS1 expression was inversely associated with HNSCC prognosis, emphasizing its potential as a prognostic marker in this malignancy. It was reported that LGALS1 was associated with lipid synthesis in adipocytes by activating PPARγ in adipocytes (11). Qin et al. found that inhibiting LGALS1 expression using OTX008 reduced the expression of CD36 and PPAR-γ, reduced the accumulation of lipid droplets in leukemia cells, and thus inhibited tumor progression (40). Interestingly, our study also showed that shLGALS1 can significantly inhibit the expression of PPARγ and change the protein level expression of E-cadherin and Snail in HNSCC cells, which indicates that LGALS1 may promote cell migration by upregulating EMT-related pathways through lipid synthesis. Although our study showed that LGALS1 was associated with FAM in early metastasis of head and neck cancer, its exact molecular mechanism remained unclear. We speculated that LGALS1 acted as a co-activator of PPARγ after entering the nucleus, directly enhancing its transcriptional activity, thereby up-regulating FASN, ACC and other fat-synthesis genes; or up-regulating CD36 expression, accelerating the cell uptake of circulating free fatty acids, and then inducing T-cell exhaustion through lipid enrichment to accelerate tumor metastasis.

This study still has some limitations. First, the sample size of lymph nodes in the scRNA-seq data is relatively small, which may lead to limited research results. The sample size needs to be increased to verify the results of this study. Secondly, the lack of metastatic lymph node data in ST may not reflect the spatial distribution characteristics of the three tumor subclusters in metastatic lymph nodes. Finally, lipidomics and rescue experiments will be used to further clarify the specific mechanism of how LGALS1 regulates HNSCC metastasis through FAM in the future.

## 5 Conclusion

Our results identified three tumor subclusters for the first time, revealing the dynamic evolution and metabolic changes of head and neck cancer cells during early dissemination. In addition, LGALS1 may serve as a novel target for fatty acid metabolism for the treatment and prognosis of head and neck cancer. This study provides new ideas for the cellular heterogeneity and molecular mechanisms of head and neck cancer, and provides a new direction for the future targeted treatment of head and neck cancer.

## Data Availability

The original contributions presented in the study are included in the article/**Supplementary Material**. Further inquiries can be directed to the corresponding author.

## Supplementary material

Supplementary Figure 1 Box plots showed the proportions of different cells in the original tumor and lymph node metastases.

Supplementary Figure 2 Identification of tumor subclusters and InferCNV analysis. **(A)** CNV assessment of epithelial cell clusters. **(B)** Box plot showing CNV scores of epithelial cell clusters. P values were calculated using two-sided Wilcoxon rank-sum test with Benjamini–Hochberg correction. **(C)** Dot plots of dynamic expression of key genes in different pathway. **(D)** Correlation heat map of key gene expression in different pathways.

Supplementary Figure 3 ST characteristics of HNSCC based on GSE208253 dataset. **(A)** SPOTlight map of overlap between scRNA-seq-identified three tumor subclusters and ST identified spot clusters. **(B)** SPOTlight map of overlap between scRNA-seq-identified T cell clusters and ST identified spot clusters. **(C)** Violin plots of the FAM source in 12 clusters. **(D)** FAM source feature plots from ST generated using the GSVA. ST, Spatial transcriptomics; FAM, Fatty acid metabolism.

Supplementary Figure 4 Cell states, EMT source, and FAM source feature plots from ST generated using SpaCET. ST, Spatial transcriptomics.

Supplementary Figure 5 Analyses of FAM-associated hub gene and validation of the effect of LGALS1 on HNSCC progression. **(A)** Kaplan-Meier survival curves revealing the correlation between ALDH3A1 expression and HNSCC prognosis. **(B)** Bar plots that LGALS1 was successfully knocked down. **(C)** Wound healing assay showed that knockdown of LGALS1 decreased HNSCC cell migration. **(D)** Bar plots that low expression of LGALS1 may affect the expression of Lipid or EMT related proteins. **(E)** Bar plots that LGALS1 was successfully overexpressed. **(F)** Bar plots showed that high expression of LGALS1 may affect the expression of EMT related proteins. FAM, Fatty acid metabolism; HNSCC, Head and neck squamous cell carcinoma; *, p<0.05; **, P<0.01.

Supplementary Figure 6 SPOTlight map of overlap between scRNA-seq-identified three tumor subclusters and ST identified spot clusters.
