# Supplementary figures and images for: Integrated analysis of single cell and spatial transcriptomics revealed a metastasis mechanism mediated by fatty acid metabolism in lymph nodes of head and neck cancer
Source: Front Immunol. 2025 Aug 13;16:1614498. doi: 10.3389/fimmu.2025.1614498 (PMC12380577; doi:10.3389/fimmu.2025.1614498)

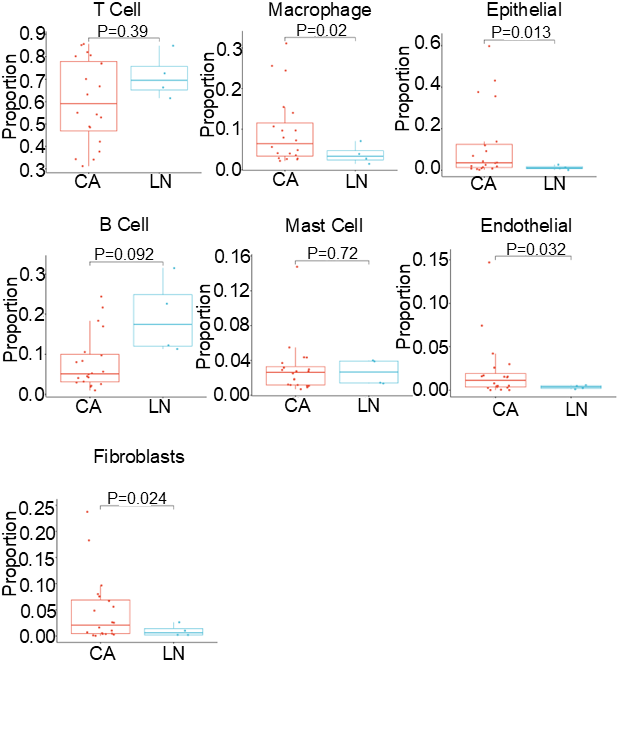

Supplement: Supplementary Figure 1 — Box plots showed the proportions of different cells in the original tumor and lymph node metastases. [file Image1.tif]

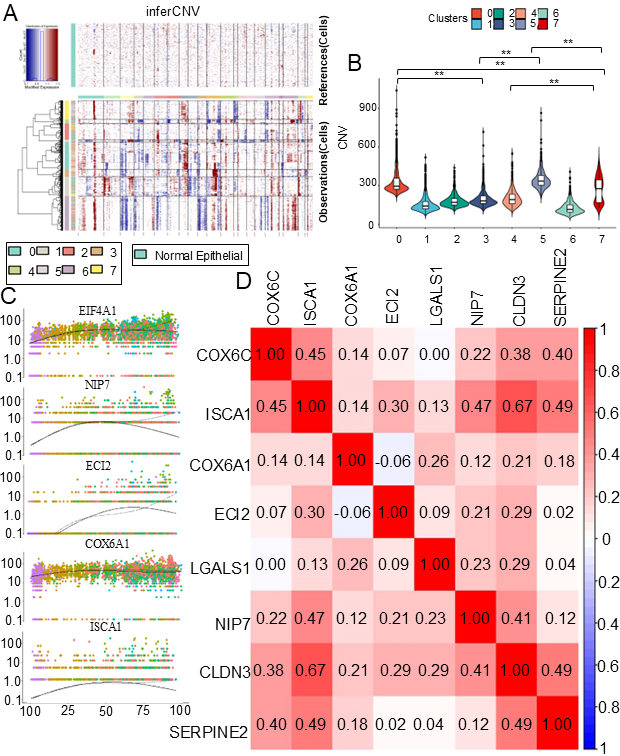

Supplement: Supplementary Figure 2 — Identification of tumor subclusters and InferCNV analysis. (A) CNV assessment of epithelial cell clusters. (B) Box plot showing CNV scores of epithelial cell clusters. P values were calculated using two-sided Wilcoxon rank-sum test with Benjamini–Hochberg correction. (C) Dot plots of dynamic expression of key genes in different pathway. (D) Correlation heat map of key gene expression in different pathways. [file Image2.tif]

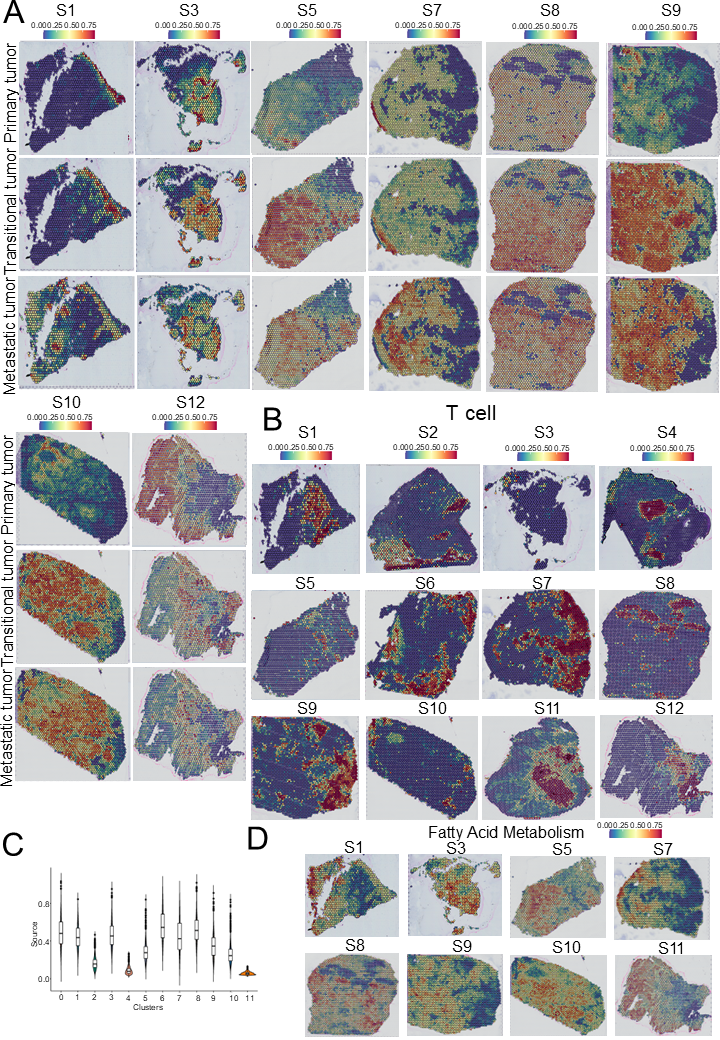

Supplement: Supplementary Figure 3 — ST characteristics of HNSCC based on GSE208253 dataset. (A) SPOTlight map of overlap between scRNA-seq-identified three tumor subclusters and ST identified spot clusters. (B) SPOTlight map of overlap between scRNA-seq-identified T cell clusters and ST identified spot clusters. (C) Violin plots of the FAM source in 12 clusters. (D) FAM source feature plots from ST generated using the GSVA. ST, Spatial transcriptomics; FAM, Fatty acid metabolism. [file Image3.tif]

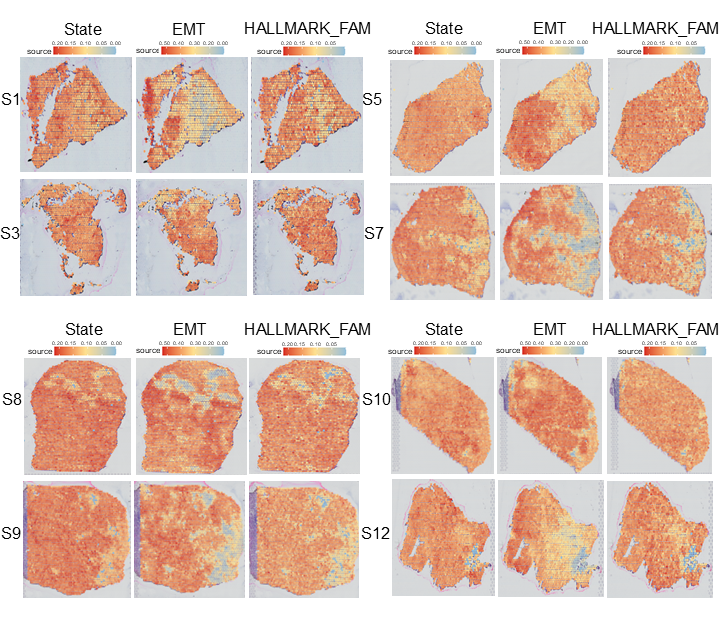

Supplement: Supplementary Figure 4 — Cell states, EMT source, and FAM source feature plots from ST generated using SpaCET. ST, Spatial transcriptomics. [file Image4.tif]

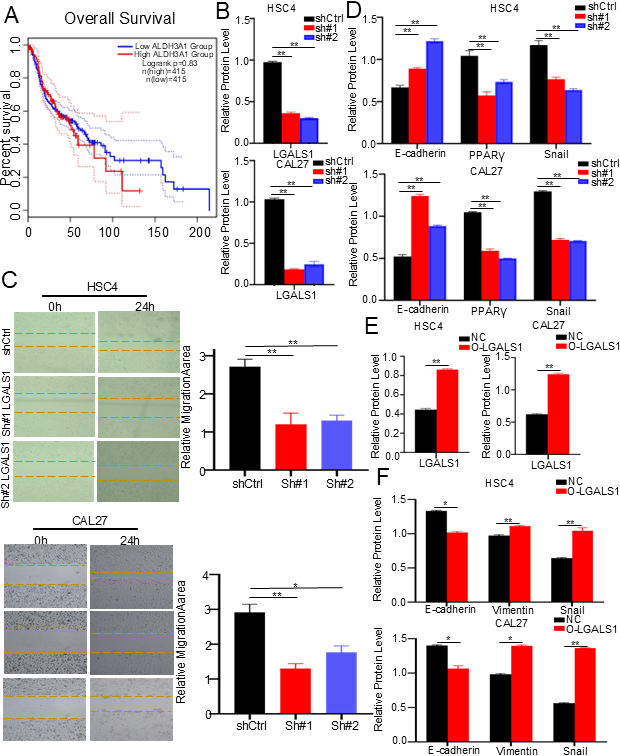

Supplement: Supplementary Figure 5 — Analyses of FAM-associated hub gene and validation of the effect of LGALS1 on HNSCC progression. (A) Kaplan-Meier survival curves revealing the correlation between ALDH3A1 expression and HNSCC prognosis. (B) Bar plots that LGALS1 was successfully knocked down. (C) Wound healing assay showed that knockdown of LGALS1 decreased HNSCC cell migration. (D) Bar plots that low expression of LGALS1 may affect the expression of Lipid or EMT related proteins. (E) Bar plots that LGALS1 was successfully overexpressed. (F) Bar plots showed that high expression of LGALS1 may affect the expression of EMT related proteins. FAM, Fatty acid metabolism; HNSCC, Head and neck squamous cell carcinoma; *, p<0.05; **, P<0.01. [file Image5.tif]

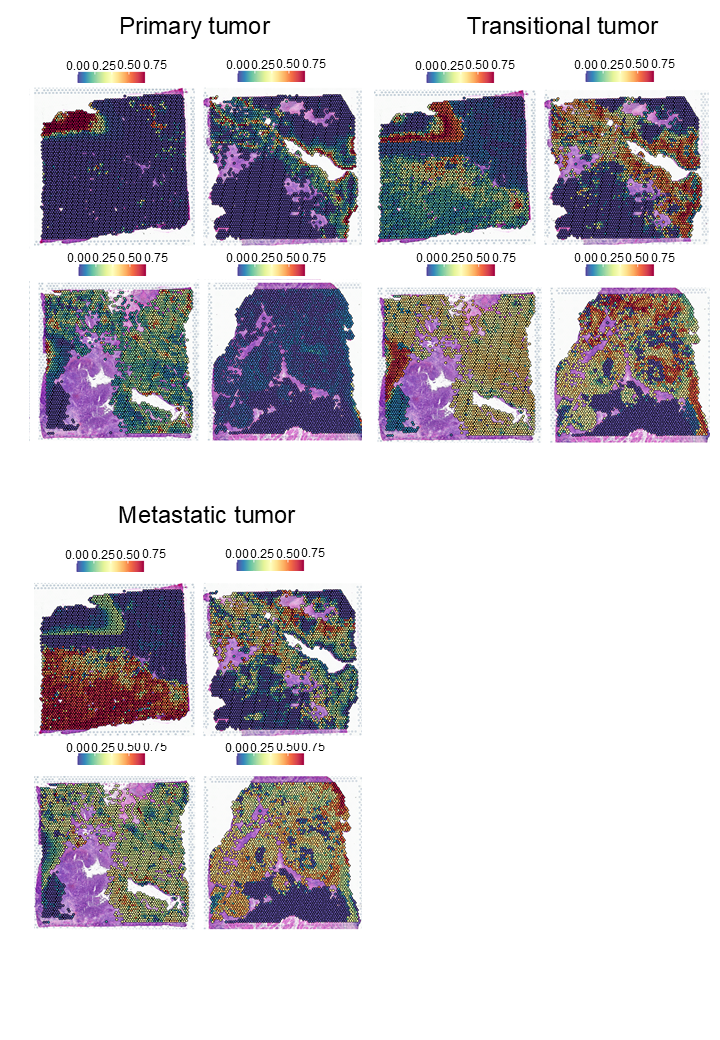

Supplement: Supplementary Figure 6 — SPOTlight map of overlap between scRNA-seq-identified three tumor subclusters and ST identified spot clusters. [file Image6.tif]
