# Supplementary material for: Integrated analysis of single cell and spatial transcriptomics revealed a metastasis mechanism mediated by fatty acid metabolism in lymph nodes of head and neck cancer
Source: Front Immunol. 2025 Aug 13;16:1614498. doi: 10.3389/fimmu.2025.1614498 (PMC12380577; doi:10.3389/fimmu.2025.1614498)
Supplement: Supplementary file 7 [file DataSheet1.docx]

Raw data link: <https://www.jianguoyun.com/p/DXUujs4Q-tTVCRiJv_IFIAA>
