# Supplementary material for: Integrated analysis of single cell and spatial transcriptomics revealed a metastasis mechanism mediated by fatty acid metabolism in lymph nodes of head and neck cancer
Source: Front Immunol. 2025 Aug 13;16:1614498. doi: 10.3389/fimmu.2025.1614498 (PMC12380577; doi:10.3389/fimmu.2025.1614498)
Supplement: Supplementary file 8 [file Table1.docx]

Table S1 Clinical sample information

| Total no. of sample | 58 |
| --- | --- |
| Age (mean ± SD) | 52±11 |
| Gender |  |
| Female | 6 |
| Male | 52 |
| Tissue |  |
| Normal | 19 |
| Tumor | 39 |
| Grade |  |
| 1 | 25 |
| 2 | 9 |
| 3 | 4 |
| Stage |  |
| I | 0 |
| II | 6 |
| III | 14 |
| IV | 17 |
| T |  |
| 1 | 0 |
| 2 | 23 |
| 3 | 9 |
| 4 | 5 |
| Lymph node metastasis |  |
| Yes | 29 |
| No | 10 |

Table S2. IHC scores of LGALS1 expressions in HNSCC. p values were determined using T test.

| Characteristics | n | | | LGALS1 level | P‑value |
| --- | --- | --- | --- | --- | --- |
| Tissues | | |  |  | 0.0107 |
| Tumor/LN metastic(-) | | | 29 | 3.25±0.431 |  |
| Tumor/LN metastic(+) | | | 10 | 4.79±0.564 |  |
| Lymph node metastasis | |  | |  | 0.0314 |
| No | | | 29 | 4.31±0.407 |  |
| Yes | | | 10 | 5.27±0.623 |  |
