# Supplementary material for: Integrated analysis of single cell and spatial transcriptomics revealed a metastasis mechanism mediated by fatty acid metabolism in lymph nodes of head and neck cancer
Source: Front Immunol. 2025 Aug 13;16:1614498. doi: 10.3389/fimmu.2025.1614498 (PMC12380577; doi:10.3389/fimmu.2025.1614498)
Supplement: Supplementary file 9 [file Table2.docx]

Table S2. IHC scores of LGALS1 expressions in HNSCC. p values were determined using T test.

| Characteristics | n | | | LGALS1 level | P‑value |
| --- | --- | --- | --- | --- | --- |
| Tissues | | |  |  | 0.0107 |
| Tumor/LN metastic(-) | | | 29 | 3.25±0.431 |  |
| Tumor/LN metastic(+) | | | 10 | 4.79±0.564 |  |
| Lymph node metastasis | |  | |  | 0.0314 |
| No | | | 29 | 4.31±0.407 |  |
| Yes | | | 10 | 5.27±0.623 |  |
